# Addressing micronutrient deficiencies and vitamin metabolism disorders in pediatric pulmonary hypertension: a strategic proposal for low- and middle-income countries

**DOI:** 10.3389/fcvm.2026.1900356

**Published:** 2026-09-24

**Authors:** Mattia Pasquinucci, Marco Scaglione, Sofia Mezzalira, Luisa Bonsembiante, Mariaelena Zaffina, Pietro Pogacnik, Sara Bondanza, Ramona Marini, Maria E. Derchi, Davide Meneghesso

**Affiliations:** 1 Department of Pediatrics, AULSS 7 Pedemontana, San Bassiano Hospital, Bassano del Grappa, Italy; 2 Department of Pediatric Metabolic Diseases, Hôpital Universitaire Necker-Enfants Malades, AP-HP, Université Paris Cité, Paris, France; 3 Department of Pediatrics and Women’s Health, University of Padua, Padua, Italy; 4 Department of Pediatric Cardiology, IRCCS Istituto Giannina Gaslini, Genoa, Italy; 5 Università degli Studi Gugliemo Marconi, Roma, Italy

**Keywords:** congenital heart disease, micronutrient deficiencies, pediatric pulmonary hypertension, scurvy, thiamine deficiency, hyperhomocysteinemia, malnutrition, iron deficiency

## Abstract

**Background:**

Pediatric pulmonary hypertension (PH) is traditionally managed under a strict hemodynamic paradigm, often overlooking systemic metabolic and nutritional drivers. This review introduces the “Cellular Deprivation Model” to evaluate how specific micronutrient deficiencies and metabolic blocks accelerate pulmonary vasculopathy.

**Methods:**

A systematic, PRISMA-compliant search was conducted in PubMed/MEDLINE for literature published between January 2000 and April 2026. Keywords intersected pediatric PH with malnutrition, avitaminosis, iron deficiency, and inborn errors of metabolism. Out of 401 unique records, 115 articles were synthesized across clinical and mechanistic tracks.

**Results:**

Acquired deficiencies (including ascorbic acid, thiamine, iron, folate, and cobalamin deficiencies) and inherited disorders of vitamin metabolism (such as Cobalamin C deficiency and folate metabolism disorders) converge on common pathophysiological pathways: the hypoxia-inducible factor (HIF) axis, causing normoxic pseudo-hypoxia, and endothelial nitric oxide synthase (eNOS) uncoupling via tetrahydrobiopterin depletion. In low- and middle-income countries (LMICs), these cellular blocks form a positive socio-hemodynamic feedback loop with congenital heart disease, accelerating vascular media hypertrophy and reducing surgical operability. Strikingly, these nutrition-driven vascular phenotypes demonstrate rapid, complete reversibility upon metabolic rescue.

**Conclusion:**

Standardized biochemical testing for micronutrients faces severe economic and cold-chain barriers in resource-limited settings. We propose a pragmatic shift toward universal empirical metabolic pre-conditioning—utilizing standardized, low-cost iron, vitamin C, thiamine, and folate regimens—for at-risk pediatric populations. This strategy aims to bypass advanced diagnostic delays, expand surgical operability windows, and mitigate perioperative mortality.

## 1 Introduction

Pediatric pulmonary hypertension (PH) remains a complex, life-threatening condition characterized by progressive pulmonary vascular remodeling, elevated pulmonary vascular resistance, and eventual right ventricular failure (1). Current clinical guidelines from the European Society of Cardiology (ESC), the European Respiratory Society (ERS), and the Cologne Consensus Conference classify pediatric PH primarily based on structural, congenital, or idiopathic etiologies, outlining standardized vasodilator and supportive therapies (1, 2). While these frameworks have significantly improved clinical algorithms in high-resource settings, they operate predominantly under a mechanical and hemodynamic paradigm, often leaving a critical diagnostic and therapeutic blind spot regarding systemic metabolic and nutritional drivers of pulmonary vasculopathy (2).

Emerging translational evidence suggests that the integrity of the pulmonary endothelium is highly dependent on precise micronutrient homeostasis. For instance, disturbances in vitamin B pathways are increasingly recognized as early cardiovascular risk factors in pediatric and adolescent populations (3). Elevated levels of plasma homocysteine (hyperhomocysteinemia) serve as a potent indicator of systemic endothelial dysfunction, triggering oxidative stress and reducing the bioavailability of nitric oxide (NO) within the vascular bed (4).

The pathogenic relevance of this metabolic axis is further supported by inborn errors of cobalamin metabolism. Children with cobalamin C (cblC) deficiency, causing combined methylmalonic aciduria and homocystinuria with hyperhomocysteinemia, may present with structural heart defects and can develop a severe and potentially fatal form of precapillary pulmonary hypertension and pulmonary microangiopathy (5, 6).

Importantly, severe acquired micronutrient deficiencies can reproduce the same aggressive pulmonary vascular phenotype observed in inherited cobalamin metabolism disorders (7, 8).

Recent clinical reports have documented an unexpected rise in precapillary PH and right-sided heart failure secondary to severe ascorbic acid deficiency (scurvy) in both developmentally typical children and those with restrictive dietary patterns, such as autism spectrum disorder (ASD) (7, 8, 10). In these cases, the loss of vitamin C disrupts endothelial oxygen sensing and collagen cross-linking, precipitating acute right-sided failure and cardiogenic shock (9, 10). Similarly, acute thiamine deficiency can trigger fulminant infantile beriberi, characterized by a rapid, catastrophic pulmonary hypertensive crisis and lactic acidosis (11). Crucially, both scurvy-induced and beriberi-induced PH demonstrate an extraordinary clinical feature: they are rapidly and completely reversible upon targeted, empirical metabolic replenishment (7, 9, 11).

Despite the profound clinical implications of these findings, a unified framework connecting vitamin metabolism disorders, nutritional deficiencies, and pulmonary vascular remodeling is currently lacking. This gap is particularly evident in low- and middle-income countries (LMICs). As foundational literature on global health has highlighted, managing pediatric PH in these regions presents unique and severe challenges (12); here, malnutrition and delayed surgical repair of congenital heart shunts frequently overlap, accelerating vascular injury. The epidemiological scale of this challenge is staggering. Recent analyses of the Global Burden of Disease demonstrate that childhood nutritional deficiencies remain a massive public health crisis, with Sub-Saharan Africa and South Asia bearing the highest incidence and mortality burdens (13). Globally, it is estimated that 41.7% of children under 5 years of age are iron deficient, while Vitamin A deficiency affects approximately 190 million preschool children (13).

This review aims to bridge this divide by introducing the “Cellular Deprivation Model.” By exploring the molecular convergence of iron, vitamin C, thiamine, and folate deficiencies on the hypoxia-inducible factor (HIF) pathway and endothelial nitric oxide synthase (eNOS) coupling, we map the “synergistic hits” that drive pulmonary vascular remodeling. Ultimately, we present a pragmatic, low-cost proposal for universal empirical metabolic pre-conditioning, designed to bypass advanced diagnostic barriers and optimize clinical outcomes in resource-limited and high-risk pediatric populations. The rationale for this targeted selection is threefold. First, mechanistically, their cellular deprivation directly disrupts the endothelial Hypoxia-Inducible Factor (HIF) axis and endothelial nitric oxide synthase (eNOS) coupling. Second, clinically, their severe deficiency precipitates acute, catastrophic, yet rapidly reversible pulmonary vasculopathy. Finally, from a global health perspective in LMICs, the selected water-soluble vitamins possess exceptionally wide pharmacological safety margins compared to fat-soluble vitamins, making universal empirical supplementation both safe and feasible in resource-constrained, unmonitored settings.

## 2 Methods

### 2.1 Search strategy and information sources

To ensure a comprehensive and reproducible mapping of the literature, a systematic search was performed on the PubMed/MEDLINE database. The search strategy was designed to capture the intersection between pediatric pulmonary hypertension (PH) and nutritional or metabolic cellular deprivation. The search strings utilized a combination of Medical Subject Headings (MeSH) terms and free-text keywords, including: (i) PH-related terms: “Hypertension, Pulmonary”, “Pulmonary Arterial Hypertension”, “Cor Pulmonale”. (ii) Nutrition and Metabolism-related terms: “Malnutrition”, “Deficiency Diseases”, “Avitaminosis”, “Iron Deficiency”, “Scurvy”, “Vitamin B12 Deficiency”, “Folic Acid Deficiency”, “Homocystinuria”, “Metabolism, Inborn Errors”. (iii) Population filters: “Child”, “Pediatrics”, “Infant”, “Adolescent”. The search was limited to studies published in English between January 1st, 2000 and April 30th, 2026. However, an explicit protocol exception was made to include seminal non-English literature fully detailing rare metabolic defects. To mitigate language bias and ensure the inclusion of critical international evidence, Artificial Intelligence (AI) tools (Gemini, Google) were utilized exclusively for the translation of non-English full-text articles into English during the data extraction phase. The authors carefully reviewed all AI-translated data against the original tables and English abstracts to guarantee scientific accuracy and clinical fidelity prior to inclusion in the review.

### 2.2 Study selection and eligibility criteria

The selection process followed a multi-step screening protocol managed through the Rayyan intelligent systematic review platform. Two investigators independently screened the titles and abstracts of all retrieved records. The initial search yielded 537 records. After the automatic and manual removal of 136 duplicates, 401 unique records were screened by title and abstract (Figure 1). Any disagreements between the two independent reviewers regarding study eligibility were resolved through discussion and consensus, or by consulting a third senior author. Inclusion Criteria were: (i) Pediatric populations (0–18 years). (ii) Documented PH, defined as a mean pulmonary arterial pressure (mPAP) >20 mmHg, directly measured via right heart catheterization (RHC) or estimated via echocardiography. (iii) Presence of nutritional deficiencies (intake-related, malabsorption, or metabolic blocks). Exclusion Criteria were considered the description of Persistent Pulmonary Hypertension of the Newborn (PPHN), Primary mitochondrial respiratory chain defects (e.g., TMEM70 deficiency) and lysosomal storage diseases (e.g., Mucopolysaccharidosis, Gaucher), as the underlying pathophysiology is not primarily nutritional or cofactor-dependent. Adult-only clinical studies were excluded unless providing essential mechanistic evidence.

**Figure 1:**
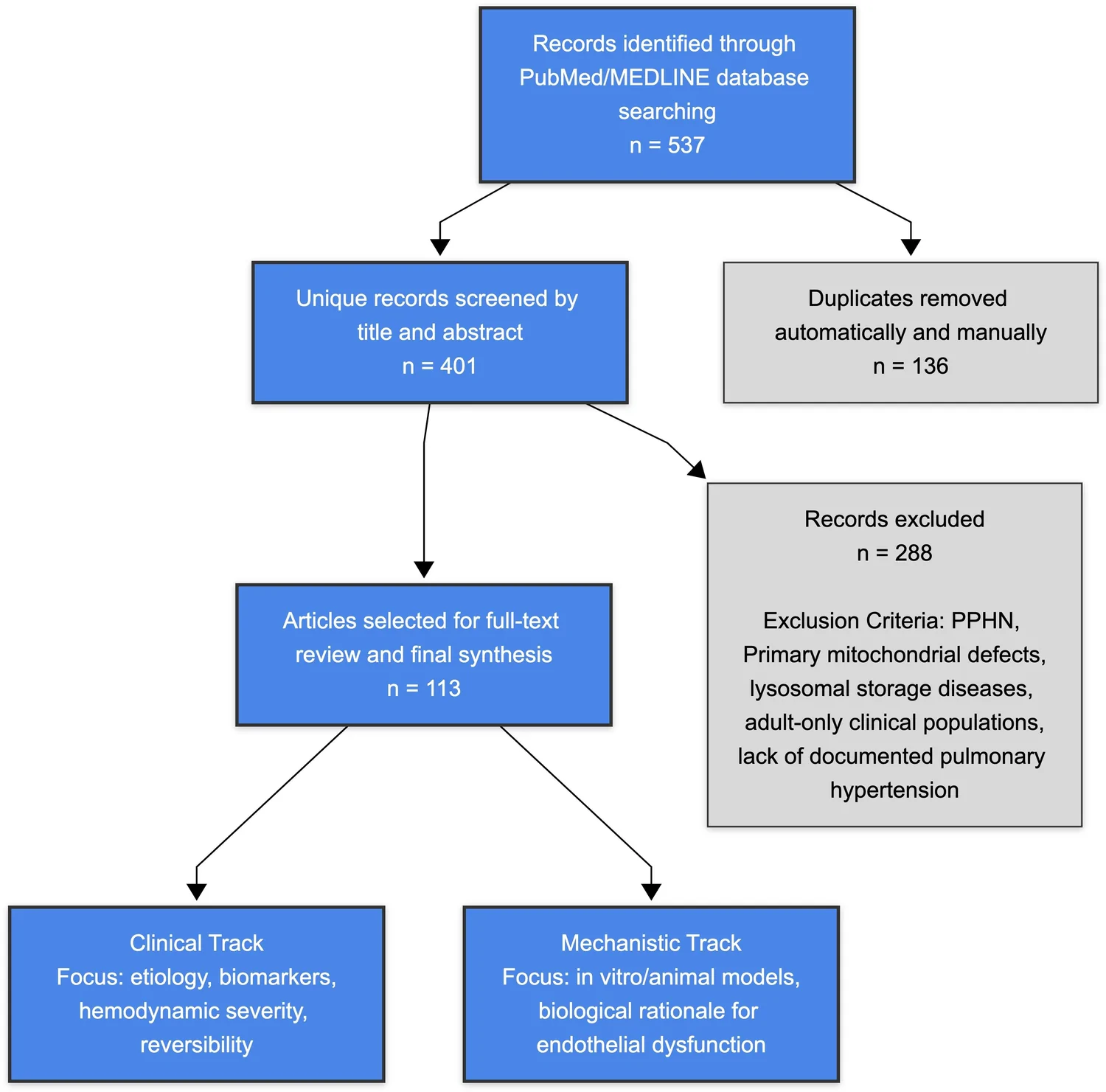
PRISMA flow diagram of the literature search and study selection process.

### 2.3 Data extraction and synthesis

A total of 113 articles were selected for full-text review. Data extraction was performed independently by two reviewers using a standardized extraction form. Following a “dual-track” methodology, the evidence was synthesized into two distinct categories:

1. Clinical Track: Extraction of clinical data from case reports and case series, focusing on etiology (intake vs. malabsorption), biochemical markers (e.g., iron, B12, homocysteine, vitamin C), hemodynamic severity, and the degree of reversibility following nutritional rescue.
2. Mechanistic Track: Review of basic science and translational studies (*in vitro* and animal models) to provide the biological rationale for the vascular endothelial dysfunction observed in the clinical cases.

The results were structured to highlight the “vicious cycle” between structural heart disease and nutritional status, particularly in resource-limited settings.

## 3 Molecular deficiencies

### 3.1 Scurvy (vitamin C deficiency)

Although historically considered an obsolete affliction associated with long maritime voyages, scurvy has recently re-emerged as a potent and under-recognized cause of reversible pulmonary hypertension (PH) in modern clinical practice (8–10). In industrialized nations, this nutritional deficiency is primarily observed in children with neurodevelopmental conditions such as Autism Spectrum Disorder (ASD) or Avoidant/Restrictive Food Intake Disorder (ARFID), who exhibit extreme food selectivity (14, 15). These patients often adhere to restricted “beige diets” limited to processed carbohydrates, milk, or sweets, with a total exclusion of fresh fruits and vegetables for durations exceeding one to 3 months (16, 17). Sociocultural beliefs can further exacerbate this risk; for instance, in some Mediterranean regions, misconceptions that citrus fruits cause pediatric cystitis or gastrointestinal toxicity when combined with milk have been documented as significant barriers to adequate intake (14).

The hemodynamic signature of scurvy-associated PH is characterized by a severe pre-capillary vasculopathy that frequently culminates in acute-on-chronic right heart failure (RHF) (15). Analyses of reported cases indicate a mPAP of 48 mmHg at presentation, with some patients exhibiting systolic pressures that reach systemic or even supra-systemic levels (18–20). This elevation is driven by a massive increase in pulmonary vascular resistance (PVR), which can reach median values nine times higher than normal physiological limits, with reported peaks as high as 41 Wood Units (21–23). Echocardiographic evaluations typically demonstrate significant right ventricular (RV) dilation driven by the abrupt increase in pulmonary afterload. As the RV attempts to overcome this massive resistance, ventriculo-arterial uncoupling occurs; this mechanical exhaustion is primarily reflected in the reduction of the tricuspid annular plane systolic excursion (TAPSE), indicating a failure of the RV's longitudinal contractile reserve. Furthermore, as the right-sided pressure equals or surpasses left ventricular (LV) pressure, adverse ventricular interdependence forces the interventricular septum to bow leftward. This paradoxical septal motion flattens the LV cavity in the short-axis view (producing the classic “D-sign”), which is a stark indicator of imminent low cardiac output and hemodynamic collapse (19, 23). In several clinical instances, the resultant RV pressure overload has been shown to cause the functional opening of a patent foramen ovale (PFO), inducing a right-to-left shunt and profound, refractory hypoxemia that can mimic primary cyanotic cardiac defects (24–26).

Undiagnosed scurvy poses a significant perioperative risk, as chronic vitamin C depletion is associated with catastrophic cardiovascular collapse during general anesthesia or sedation (16, 27). Multiple reports have documented children suffering sudden pulseless electrical activity (PEA) arrest during routine diagnostic procedures such as endoscopies or biopsies (21, 27). This hemodynamic lability is often compounded by a markedly blunted response to conventional pulmonary vasodilators—including sildenafil, bosentan, and inhaled nitric oxide—which frequently remain ineffective until the underlying metabolic deficiency is corrected (16, 20, 27). Biochemically, vitamin C serves as a mandatory cofactor for dopamine β-hydroxylase; therefore, its deficiency impairs catecholamine synthesis, resulting in autonomic dysfunction and severe, vasopressor-resistant hypotension (10, 18).

The hallmark of PH in this context is the dramatic “metabolic rescue” observed upon ascorbic acid repletion. Significant clinical and hemodynamic improvements are typically documented within 24–48 h of initiating therapy (9–11, 15, 27–39). Complete resolution of PH and the normalization of RV function are generally achieved between 1 week and 3 months (9, 11, 22, 23, 25). Furthermore, emerging evidence suggests that severe vitamin C deficiency may activate the T-helper 17 (Th17) inflammatory pathway, significantly elevating levels of IL-6, IL-17A, and IL-23, which contributes to perivascular inflammation and endothelial damage (22). Data are summarised in Table 1.

**Table 1:** Clinical and hemodynamic profiles of pulmonary hypertension in scurvy.

Paper	Year	Cases (*n*)	Vitamin C levels (μmol/L)	PH diagnosis	Maximum sPAP (mmHg)	Maximum mPAP (mmHg)	Heart failure	Cardioactive treatment	Time to resolution (days)	Other deficiencies	Anemia
Pasquinucci et al. (8)	2026	1	<2 μmol/L	Cardiac US	45	35	No	No	7	Yes (folate, vitamin D)	Yes
Satawiriya et al. (9)	2023	2	Undetectable	Heart catheterisation	29, 66	20, 50	Yes	Yes	14	Yes	Yes
Abe et al. (10)	2023	1	Undetectable	Cardiac US	NR	NR	Yes	no	30	No	Yes
Kurnick et al. (15)	2023	1	Undetectable	Heart catheterisation	70	48	Yes	Yes	23	No	Yes
Gayen et al. (16)	2020	1	Undetectable	Heart catheterisation	76	41	No	No	150	Yes (vitamin D)	Yes
Ghulam Ali and Pepi (17)	2018	1	NR	Cardiac US	97	NR	No	No	14	NR	Yes
Dean et al. (19)	2018	1	<5	Cardiac US	NR	NR	Yes	Yes	180	Yes (vitamin E, vitamin A, vitamin B6)	Yes
Tan et al. (20)	2020	1	<5	Cardiac US	NR	NR	Yes	No	10	NR	NR
Ferreira et al. (21)	2019	1	0.8	Cardiac US	61	NR	No	No	NR	Yes (vitamin B12, folic acid)	Yes
Shah et al. (22)	2021	1	NR	Cardiac US	NR	NR	No	No	14	Yes (thiamine)	Yes
Kang et al. (23)	2024	1	Undetectable	Cardiac US	NR	32	Yes	No	7	Yes (folic acid)	Yes
Frank et al. (24)	2019	1	Undetectable	Cardiac US	NR	NR	No	Yes	2	Yes (vitamin D)	Yes
Berra et al. (25)	2025	1	2.1	Cardiac US	NR	32	Yes	Yes	7	Yes (thiamine)	Yes
Silberkasten (26)	2025	1	Undetectable	Cardiac US	NR	NR	Yes	No	21	Yes (Vitamin D, vitamin B12, folic acid)	Yes
Sakamornchai et al. (27)	2022	2	Undetectable	Cardiac US	NR	NR	Yes	Yes	13, 5	Yes (Vitamin D)	Yes
Flanagan et al. (28)	2025	1	NR	Cardiac US	46	34	Yes	Yes	6	NR	Yes
Ichiyanagi et al. (29)	2019	1	Undetectable	Cardiac US	98	77	Yes	Yes	14	Yes (thiamine)	NR
Ayyad et al. (30)	2025	2	Undetectable	NR	NR	NR	Yes, No	Yes	8	NR	Yes
Zavaleta and Burt (31)	2020	1	Undetectable	NR	NR	NR	No	No	7	NR	Yes
Nariai et al. (32)	2022	1	Undetectable	Cardiac US	NR	NR	Yes	Yes	35	Yes (thiamine)	Yes
Quinn et al. (33)	2022	1	NR	Cardiac US	NR	NR	Yes	Yes	42	Yes (vitamin A, vitamin D, zinc, thiamine)	Yes
Somech et al. (35)	2026	1	Undetectable	Cardiac US	NR	NR	Yes	Yes	4	Yes (vitamin B1, vitamin B12)	Yes
Azar et al. (36)	2023	1	Undetectable	Heart catheterisation	NR	41	Yes	Yes	NR	NR	NR
Toscano et al. (39)	2025	2	Undetectable	Cardiac US	30, 80	NR	No	No	3	NR	NR

Summary of compiled prospective cohorts, retrospective case series, and case reports mapping the phenotypes of cardiovascular vitamin C deficiency. Data regarding vitamin C levels, hemodynamic pressures, and time to resolution are reported as mean ± standard deviation, median (interquartile range), or absolute values based on the original source data. mPAP, mean pulmonary artery pressure; NR, not reported in the original article; sPAP, systolic pulmonary artery pressure; US, ultrasound (echocardiography).

### 3.2 Thiamine deficiency (vitamin B1)

Thiamine (vitamin B1) plays a fundamental role in cellular energy metabolism, acting as an essential cofactor for pyruvate dehydrogenase, the enzyme responsible for the conversion of pyruvate into acetyl-CoA, a key substrate of the Krebs cycle (11, 40). Unlike other micronutrients, thiamine has a rapid turnover rate and limited body storage—approximately 30 mg—leading to the depletion of systemic reserves in as little as two weeks without continuous dietary intake (41). This nutritional gap remains a significant public health challenge in specific geographic regions, especially in South and Southeast Asia, where the consumption of polished white rice and sociocultural dietary taboos during the peripartum period frequently deprive exclusively breastfed infants of adequate thiamine during the first few months of life (42, 43). In these settings, the deficiency is primarily nutritional rather than genetic, as affected infants typically possess wild-type thiamine transporter genotypes (SLC19A2 and SLC19A3) (44).

The clinical spectrum of infantile thiamine deficiency is classically divided into cardiovascular (wet) and neurological (dry) beriberi (45). A fulminant and hemodynamically catastrophic variant, known as Shoshin beriberi, presents as sudden cardiovascular collapse characterized by hypotension, cardiogenic shock, and profound lactic acidosis (46). More recently, Thiamine-Responsive Acute Pulmonary Hypertension (TRAPH) has gained recognition as a distinct phenotype, frequently mimicking neonatal sepsis or lower respiratory infections in infants aged 1–6 months (47).

Hemodynamically, TRAPH is characterized by severe, often supra-systemic pulmonary arterial hypertension associated with massive right heart dilation and right-to-left shunting across a PFO (42, 46). The metabolic block at the level of the pyruvate dehydrogenase complex leads to a near-universal finding of hyperlactatemia, as pyruvate is diverted towards anaerobic conversion to lactate (43, 45). Despite the severity of the presentation, the administration of parenteral thiamine induces significant reduction in pulmonary pressures typically documented within 24–48 h (41–43, 46). This recovery is facilitated by the restoration of myocardial energy production and the reduction of oxidative stress, as thiamine deficiency is associated with increased reactive oxygen species (ROS) and impaired nitric oxide (NO) bioavailability (42, 43, 48). Data are summarised in Table 2.

**Table 2:** Clinical and hemodynamic profiles of thiamine-responsive pulmonary hypertension (TRAPH) and infantile beriberi.

Paper	Year	Cases (*n*)	Thiamine levels (nmol/L)	PH diagnosis	Maximum sPAP (mmHg)	Beriberi presentation	Cardioactive treatment	Time to resolution (days)	Other deficiencies	Anemia
Samprathi et al. (11)	2021	4	NR	Cardiac US	55 (50–60)	Shoshin (2)	Yes	1	NR	Yes (3), No (1)
						Wet (2)				
Nisar et al. (40)	2024	6	27.8 ± 10.9	Cardiac US	43.0 ± 13.0	Wet (3),	No	1	NR	Yes (6)
						TRAPH (3)				
Suryakanthi et al. (41)	2022	6	13.4 (3.9–24.1)	Cardiac US	NR	Shoshin (6)	Yes	2	NR	Yes (6)
Sastry (42)	2021	238	NR	Cardiac US	73.2 ± 14.4	TRAPH (231), Shoshin (7)	Yes	1	NR	NR
Panigrahy et al. (43)	2020	4	25 (3.7–75)	Cardiac US	71 (60–82)	TRAPH (4)	Yes	6 (5–7)	NR	NR
Shenoy et al. (44)	2024	40	NR	Cardiac US	51 (40–69)	TRAPH (40)	NR	NR	NR	NR
Qureshi et al. (45)	2021	5	NR	Cardiac US	51 (mean)	TRAPH (4), Shoshin (1)	Yes	3	NR	Yes (5)
Bhat et al. (46)	2017	29	NR	Cardiac US	48	TRAPH (29)	NR	28	NR	Yes
Rao and Chandak (47)	2010	55	NR	Cardiac US	NR	Wet (55)	NR	NR	NR	No
Pache-Wannaz et al. (48)	2025	1	39.6	Cardiac US	98	TRAPH	Yes	2	Yes (Folates, Vit. C, iron)	NR

Summary of compiled pediatric cohorts and case reports mapping the cardiovascular phenotypes of thiamine deficiency. The “Beriberi presentation” column differentiates between classical Wet beriberi, fulminant Shoshin beriberi, and the specific TRAPH phenotype. NR, not reported in the original article; sPAP, systolic pulmonary artery pressure; TRAPH, thiamine-responsive acute pulmonary hypertension; US, ultrasound (echocardiography).

### 3.3 Methylmalonic aciduria and hyperhomocysteinemia (MMA-HC)

Combined methylmalonic aciduria and hyperhomocysteinemia (MMA-HC) is an inborn error of metabolism where pulmonary hypertension (PH) has emerged as a severe, under-recognized complication (5, 6, 49–51). Although traditionally defined by neurodevelopmental impairment, PH is increasingly recognized as the primary manifestation in both neonatal cases—where it directly mimics persistent pulmonary hypertension of the newborn (PPHN) (50)—and late-onset presentations marked by urgent dyspnea and cyanosis (49, 52). Alongside concurrent organ involvement (predominantly renal injury and macrocytic anemia), these patients display a distinct biochemical signature: extreme hyperhomocysteinemia and elevated brain natriuretic peptide (BNP) (49, 52, 53). This profile reflects a profound right ventricular pressure overload capable of precipitating rapid cardiogenic shock (49, 54). The cobalamin C (cblC) defect is the most prevalent subtype of MMA-HC associated with aggressive pulmonary vasculopathy (49, 51–57). The pathogenesis of PH in cblC deficiency is largely attributed to severe hyperhomocysteinemia, which induces direct endothelial toxicity, promotes smooth muscle cell proliferation, and triggers pulmonary microangiopathy (54, 58, 59). Despite the devastating nature of this “metabolic cor pulmonale,” the hallmark of the disease is its remarkable response to targeted therapy (49, 51, 60–62). The administration of parenteral hydroxocobalamin, combined with betaine and PH-targeted vasodilators, acts as a definitive “metabolic rescue” for many patients (49, 52, 61). Clinical stabilization often occurs within weeks, with complete echocardiographic normalization of pulmonary artery pressures typically achieved within 3–18 months (49, 51, 62–66). However, metabolic rescue is not universally successful. As reported in pediatric cohorts (50, 59, 61, 63), some patients still exhibited progressive vasculopathy or suffered fatal right ventricular failure despite high-dose hydroxocobalamin. This refractory course likely reflects irreversible structural remodeling highlighting the critical need for prompt intervention before end-stage changes occur. Data are summarised in Table 3.

**Table 3:** Summary of published literature highlighting diagnostic modalities, hemodynamic severity, and clinical outcomes following metabolic rescue in patients with combined methylmalonic aciduria and homocystinuria (MMA-HC).

Paper	Year	Cases (*n*)	Homocysteine levels (μmol/L)	PH diagnosis	Maximum sPAP (mmHg)	Heart failure	Cardioactive treatment	Time to resolution (days)	Other deficiencies	Anemia
Profitlich et al. (5)	2009	1	160	Cardiac US	NR—cor pulmonale	Yes	NR	NR	NR	NR
Qi et al. (6)	2015	5	151 ± 51	Cardiac US	Severe (3), moderate (1), mild (1)	Yes	Yes	135 (90–180)	NR	Yes (3)
Liu et al. (49)	2017	15	121.2 ± 48.2	Cardiac US	90.3 ± 23.9	Yes	Yes	90	NR	Yes (8)
Agarwal et al. (50)	2014	1	3.4	Cardiac US	42	Yes	Yes	Death	NR	NR
He et al. (51)	2025	17	110 (34–229)	Cardiac US	NR	Yes	Yes	135 (30–540)	Yes (methionin)	Yes (12)
Zhao et al. (52)	2025	9	115.94 (31.4–214.8)	Cardiac US	81 ± 17.6	Yes	Yes	270 (180–360)	NR	Yes (5)
Liu et al. (53)	2023	2	125–555	Cardiac US	NR	Yes	NR	NR	NR	Yes (2)
Liu et al. (54)	2020	4	119.5 (103–136)	Cardiac US/HRCT	NR	Yes	Yes	Below 365	NR	Yes (2)
Liu et al. (55)	2017	4	>50 (2), 193	Cardiac US	68, 81	Yes	Yes	60 (30–365)	Yes (methionin)	Yes (2)
Iodice et al. (56)	2013	1	NR	NR	NR	NR	NR	NR	NR	NR
De Simone et al. (57)	2018	1	74	Cardiac US	107	Yes	Yes	15	NR	Yes
Tang et al. (58)	2019	6	NR	HRCT	NR	Yes	Yes	NR	NR	Yes (2)
Wen et al. (60)	2020	1	155.8	Heart catheterisation	NR	Yes	Yes	90	Yes (methionin)	Yes
Gupta et al. (61)	2021	1	NR	Cardiac US	NR	Yes	Yes	death	NR	Yes
Profitlich et al. (62)	2009	1	181	Cardiac US	58	Yes	Yes	75	NR	Yes
Chioukh and Monastiri (63)	2019	1	NR	Cardiac US	NR	Yes	Yes	death	NR	NR
Liao et al. (64)	2020	1	119.99	Heart catheterisation	70	Yes	Yes	180	NR	Yes
Kömhoff et al. (59)	2014	5	NR	NR	NR	Tes	NR	Death (3)	NR	Yes

HRCT, high-resolution computed tomography; NR, not reported in the original article; sPAP, systolic pulmonary artery pressure; US, ultrasound (echocardiography).

### 3.4 Folate deficiency and homocysteine metabolism

Folate (vitamin B9) is a central regulator of vascular homeostasis, serving as a critical methyl donor in the methionine cycle and a mandatory cofactor for the maintenance of endothelial function (3, 4). While systemic folate deficiency is often observed in the context of global malnutrition, specific genetic defects in folate transport and metabolism have highlighted its independent role in the pathogenesis of pediatric PH. Hereditary folate malabsorption (HFM), caused by mutations in the *SLC46A1* gene encoding the proton-coupled folate transporter (PCFT), represents a rare but instructive model of “pure” folate-induced vasculopathy (67). Infants with HFM typically present with megaloblastic anemia, immunodeficiency, and neurological deterioration; however, severe PH has been documented as a life-threatening complication in this population (67). Similar to other metabolic forms of PH, folate-related vasculopathy exhibits remarkable reversibility (67).

The mechanistic link between folate deficiency and PH is predominantly mediated through the modulation of NO bioavailability and the regulation of homocysteine levels. Folates, particularly in the form of 5-methyltetrahydrofolate (5-MTHF), play a crucial role in preventing endothelial nitric oxide synthase (eNOS) uncoupling (68, 69). Under conditions of folate deprivation, there is a significant reduction in the recycling of 5,6,7,8-tetrahydrobiopterin (BH4), an essential co-factor for eNOS (69, 70). This biochemical shift causes eNOS to deviate from its physiological production of NO and instead generate superoxide anion radicals, thereby fostering a pro-oxidant and vasoconstrictive environment within the pulmonary circulation (70, 71). Experimental models in newborn piglets exposed to chronic hypoxia have demonstrated that folic acid supplementation, either alone or in synergy with L-citrulline, can effectively restore NO signaling, reduce arginase activity, and ameliorate pulmonary vascular remodeling (71, 72).

Furthermore, folate deficiency is a primary driver of hyperhomocysteinemia (68, 70). While mild-to-moderate hyperhomocysteinemia is a well-established pro-thrombotic risk factor for macroscopic pulmonary embolism—which notably often occurs without progressing to pulmonary hypertension in adult cohorts (73)—the pediatric pulmonary vascular phenotype described here is driven by *in situ* microvascular remodeling rather than macroscopic clots. Elevated homocysteine levels induce direct endothelial injury and, crucially, exert profound effects on the medial layer by promoting pulmonary artery smooth muscle cell (PASMC) proliferation and migration (74). Additionally, hyperhomocysteinemia decreases the expression of protective molecules like hydrogen sulfide, which normally exerts a vasodilator and anti-remodeling effect on the pulmonary arteries (68). Clinical studies in children with left-to-right shunts have identified a significant correlation between high homocysteine levels, low serum folate, and the severity of PH, suggesting that the folic acid-homocysteine axis acts as a potent “modulator” of the pulmonary vascular phenotype (68).

### 3.5 Iron deficiency: the bioenergetic and regulatory axis

Iron is a fundamental metabolic regulator whose role in pulmonary vascular homeostasis extends far beyond oxygen transport. Within the pulmonary circulation, iron serves as a mandatory cofactor for non-heme ferrous iron-dependent dioxygenases, specifically the prolyl-hydroxylases (PHD1–3) that act as the cell's primary oxygen sensors (75). Under conditions of iron deficiency, these enzymes fail to catalyze the degradation of the hypoxia-inducible factor (HIF)-α subunit, even in normoxic environments (76). This inappropriate stabilization of HIF-1α and HIF-2α triggers a “pseudo-hypoxic” transcriptional program, promoting pulmonary artery smooth muscle cell (PASMC) proliferation and vasoconstriction through mediators such as endothelin-1 (77, 78).

Clinical investigations have demonstrated that iron deficiency is remarkably prevalent in PAH, affecting up to 63% of patients with idiopathic forms (79–87). However, prevalence estimates vary significantly depending on the specific biomarker thresholds used to define deficiency (80). Elevated levels of soluble transferrin receptor (sTfR) serve as a robust biomarker for this deficiency (79, 87, 88). Furthermore, iron status appears to dictate the magnitude of the pulmonary vascular response to hypoxia (78, 81).

Historically, the regulation of iron in PH is closely tied to the bone morphogenetic protein (BMP) signaling pathway, where dysfunctional BMPR2 signaling led to the inappropriate elevation of hepcidin, sequestering iron in macrophages (functional iron deficiency) (83, 84, 87). This systemic sequestration is accompanied by local mitochondrial dysfunction and a metabolic shift toward glycolysis within the lung (83, 85). However, recent data reveal a highly complex regulatory landscape. Studies such as Theobald et al. found no significant difference in hepcidin levels or iron deficiency prevalence based on BMPR2 mutation status (89). Furthermore, Karamania et al. demonstrated that some PH patients exhibit reduced hepcidin and elevated erythropoietin (EPO) levels within lung tissue and PASMCs, pointing to profound local hypoxic signaling rather than a uniform systemic hepcidin block (90). Importantly, the relationship between iron and pulmonary hemodynamics appears to follow a delicate “U-shaped” curve. Just as deficiency drives HIF-mediated remodeling, iron excess (e.g., hemochromatosis) can also exacerbate PH (91).

Consequently, the clinical translation of iron replacement therapy remains highly controversial. Rigorous randomized controlled trials and comprehensive studies failed to demonstrate significant improvements in primary endpoints such as 6-minute walk distance (6MWD) or resting pulmonary vascular resistance (PVR) following intravenous iron therapy in adult PAH cohorts (92). However, therapeutic intervention with intravenous iron (e.g., iron carboxymaltose) has been shown to effectively bypass the hepcidin block, significantly improving secondary outcomes like exercise endurance capacity and quality of life (88). As demonstrated by Ruiter et al., these clinical benefits occur even in the absence of significant changes in resting hemodynamics, likely driven by improved oxygen handling in skeletal muscle cells (88, 93).

Furthermore, a critical pathophysiological distinction must be made regarding our target population. Adult PAH cohorts predominantly present with *functional* iron sequestration within mature vascular beds characterized by advanced, fixed remodeling. In stark contrast, pediatric populations in LMICs suffer from profound, *absolute nutritional* iron deficiency, compounded by the immense metabolic and anaplerotic demands of somatic growth. The pediatric pulmonary endothelium is functionally immature and highly reactive to stress stimuli. In these resource-limited settings, where the risk of chronic pathological hemosiderosis is negligible compared to the 41.7% global prevalence of pediatric iron deficiency (13), correcting absolute iron deficits is a physiological necessity. While its ability to reverse end-stage fibrotic remodeling remains uncertain, early metabolic rescue in children is essential to restore basic oxygen-carrying capacity, dampen pseudo-hypoxic signaling, and optimize right ventricular functional reserve before the vascular phenotype becomes fixed.

## 4 Mechanisms of combined nutrient deficiency

The pathogenesis of pediatric PH in the context of cellular deprivation is rarely the result of an isolated nutrient deficit. Instead, it emerges from a “synergistic hit” where multiple biochemical blocks converge on the pulmonary endothelium, causing a multi-level failure of vascular homeostasis (94, 95). This “Cellular Deprivation Model” encompasses acute vasomotor dysregulation, chronic structural remodeling, and systemic metabolic collapse (Figure 2) (96–106).

**Figure 2:**
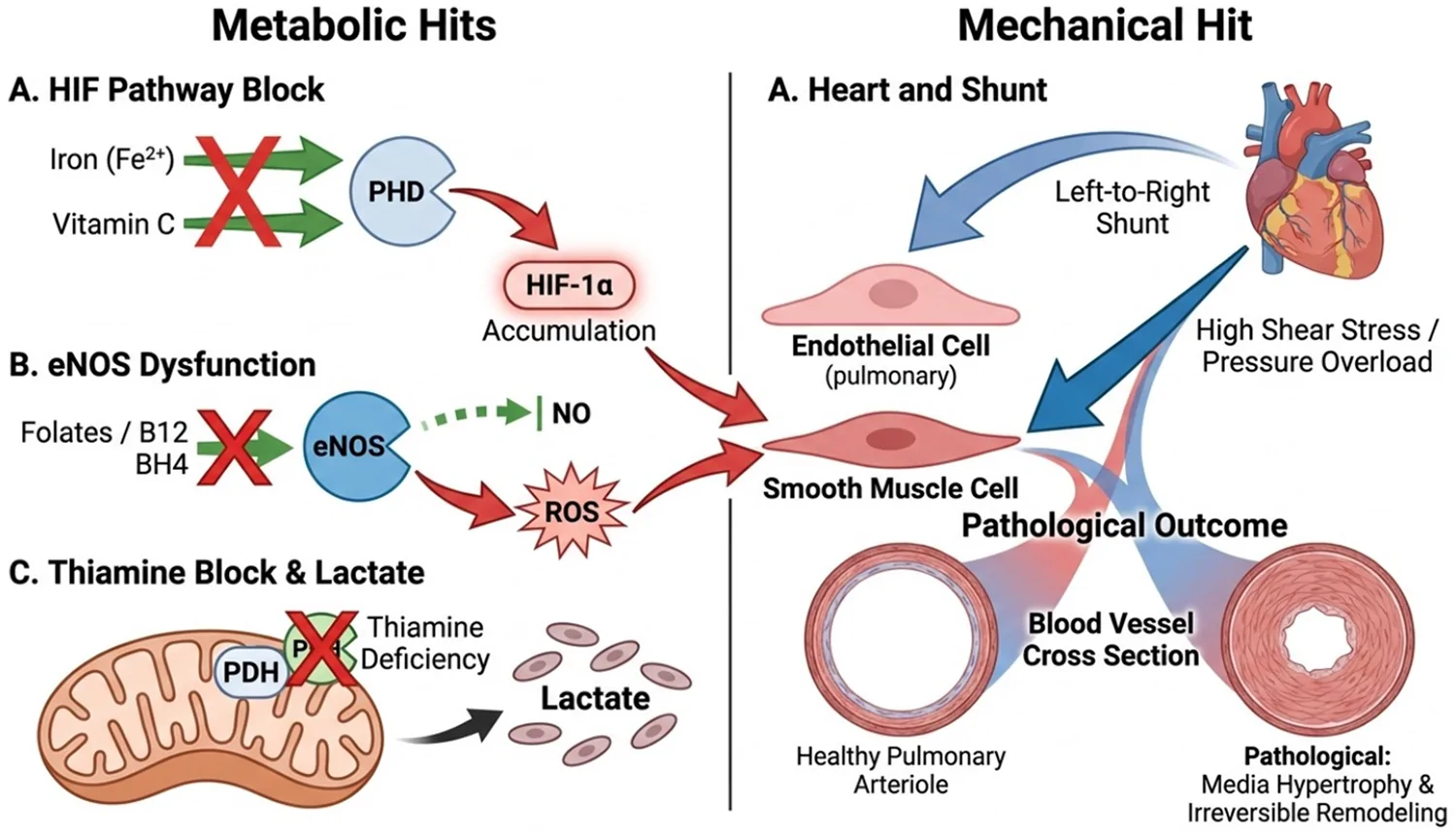
The cellular deprivation model in pediatric pulmonary hypertension.

### 4.1 HIF-hydroxylase

The molecular cornerstone of this pathology is the stabilization of hypoxia-inducible factors (HIF-1α and HIF-2α) under normoxic conditions (94, 104). The PHD enzymes, which serve as cellular oxygen sensors, are iron-dependent dioxygenases that require ferrous iron (Fe^2+^) as a catalytic center and ascorbic acid to maintain iron in its reduced state (94, 95, 111). Humans are uniquely vulnerable to this mechanism due to the evolutionary loss of the L-gulonolactone oxidase (GLO) enzyme, making vitamin C an obligatory dietary micronutrient (100). This vulnerability is further exacerbated by metabolic competition; for instance, hyperglycemia can inhibit the transport of dehydroascorbic acid into cells via facilitative glucose transporters, inducing a localized intracellular vitamin C deficiency even in the presence of adequate systemic levels (104–110).

### 4.2 Endothelial uncoupling and the folate-homocysteine axis

Vascular health relies on NO bioavailability, which is regulated by the folate-B12-homocysteine axis (101, 102). Folates (B9) and vitamin C act in concert to stabilize and recycle tetrahydrobiopterin (BH4), an essential co-factor for endothelial NO synthase (eNOS) (95, 107). Simultaneous deficiency in folates, B6, and B12 leads to the accumulation of homocysteine (HHcy), a potent marker of early endothelial dysfunction (98, 99, 111). HHcy-induced oxidative stress causes “eNOS uncoupling,” where the enzyme shifts from producing vasodilatory NO to generating superoxide radicals, fostering a pro-thrombotic and vasoconstrictive microenvironment (96, 97, 107). High-dose supplementation with folate and B6 has been shown to rapidly normalize this endothelial dysfunction, highlighting the potential for immediate clinical improvement through metabolic optimization (98, 99).

### 4.3 Systemic inflammation and developmental programming

Chronic inflammation (IL-1, IL-6) and right ventricular remodeling lead to a functional decline that extends to skeletal muscle, often presenting as “cardio-skeletal” wasting (106, 107). Genetic epidemiology suggests that certain children may be more susceptible to this metabolic hit due to variants in PAH-associated genes (e.g., *BMPR2*, *SLC19A2/3*), which can modulate the threshold for vascular injury (44, 106, 107).

Finally, nutritional insults during the “First 1000 Days” can program a “vulnerable pulmonary bed” through epigenetic mechanisms such as DNA hypermethylation (102, 103). This epigenetic “imprinting” increases susceptibility to aggressive PH when the child faces a “second hit,” such as a congenital heart shunt or high-altitude exposure, later in life (102–104). This vulnerability can be further compounded by postnatal growth restriction and intestinal dysbiosis, which have been shown to synergistically impair pulmonary vascular development via the gut-lung axis (112).

## 5 Congenital heart disease in low- and middle-income countries: a socio-hemodynamic feedback loop

### 5.1 Prevalence and determinants of undernutrition in CHD

In low- and middle-income countries, the intersection of CHD and systemic undernutrition represents a critically neglected global health crisis. Accumulating epidemiological data and meta-analyses from resource-limited regions demonstrate that over 50% of pediatric patients with CHD suffer from moderate-to-severe malnutrition at clinical presentation (113, 114). Anthropometric indices reveal a devastating combined prevalence of wasting, stunting, and underweight, ranging from 23% to over 50% across sub-Saharan African and South Asian cohorts (115, 116). As highlighted by foundational global cardiology networks (12), the underlying etiology of this systemic somatic wasting is inherently multifactorial and self-perpetuating. The severe cachexia is driven by a chronic mismatch between chronically low dietary micronutrient intake and a massive escalation in resting energy expenditure, induced by the mechanical and neurohormonal stress of myocardial volume and pressure overload (113, 116, 117). Crucially, this metabolic failure is further exacerbated by the underlying cardiopulmonary pathophysiology: systemic venous congestion and chronic hypoxemia induce mucosal edema and secondary intestinal malabsorption, severely hindering nutrient uptake (115–117). Consequently, children in these regions are trapped in a vicious cycle where malnutrition prevents surgical eligibility, while the uncorrected cardiac defect actively drives the child deeper into metabolic exhaustion.

### 5.2 Pulmonary hypertension as a driver of metabolic collapse

The progression from mild nutritional vulnerability to severe cardiac cachexia is heavily accelerated by the development of PH, establishing a pathological positive feedback loop. Hemodynamic data derived from right heart catheterization reveal a direct, linear correlation between elevated pulmonary vascular resistance and the severity of somatic deperishement (118). In acyanotic children with left-to-right shunts the onset of PH doubles the relative risk of acute wasting (114).

This metabolic collapse reaches its peak in patients exhibiting a combined phenotype of PH and chronic cyanosis, where the prevalence of severe malnutrition reaches 100% (117). Chronic hypoxemia paired with systemic venous congestion significantly compromises the integrity of the intestinal mucosal barrier, exacerbating malabsorption and inducing a cachectic state that remains refractory to standard caloric supplementation (117, 118). In these advanced stages, severe mucosal edema and systemic inflammation—which notably upregulates hepcidin, blocking enteral iron uptake—render the efficacy of oral metabolic replacement highly unpredictable. Correcting this cellular deprivation therefore requires a synergistic approach. While micronutrients act as essential cofactors to reignite endothelial enzymatic function, they must be coupled with targeted macronutrient rehabilitation. As demonstrated by Vinke et al., high-protein and anti-inflammatory nutritional interventions can significantly attenuate right ventricular and skeletal muscle wasting in PAH models (108). Therefore, our proposed micronutrient pre-conditioning is designed to complement, not replace, comprehensive protein support programs. In this context, malnutrition has emerged as a significant, independent predictor of poor prognosis and mortality in PAH patients (75). Crucially, the resulting cellular deprivation of key micronutrient cofactors (such as iron, folates, and ascorbic acid) accelerates endothelial nitric oxide synthase (eNOS) uncoupling and vascular remodeling, further worsening the underlying pulmonary hemodynamics (117, 118).

### 5.3 Socioeconomic barriers, loss of operability, and accelerated Eisenmenger syndrome

In resource-constrained environments, prolonged diagnostic and surgical delays transform anatomically correctable structural shunts into irreversible vascular pathologies. Families navigating these healthcare systems face catastrophic out-of-pocket expenses during the pre-surgical phase, frequently forcing them to sell assets or secure high-interest loans to afford palliative medical therapies and sequential monitoring (119). These severe financial barriers inevitably delay corrective surgery far beyond the therapeutic windows recommended by international guidelines (119).

The persistence of unmitigated hemodynamic shear stress on the pulmonary capillary bed accelerates arteriole muscularization and medial hypertrophy, culminating in the exceptionally early development of Eisenmenger syndrome during infancy or early childhood (113, 118). While definitive surgical correction is precluded in these advanced stages, targeted metabolic optimization offer critical translational avenues to improve survival. Correcting iron deficiency—which is highly prevalent in polycythemic cyanotic cohorts—has been shown to significantly enhance right ventricular functional reserve and exercise capacity (120). Furthermore, given the severe impairment of the endothelial NO pathway in these children, preoperative or perioperative supplementation with metabolic precursors such as L-citrulline represents a low-cost strategy to reduce the incidence of postoperative pulmonary hypertensive crises and optimize clinical outcomes in complex surgical settings (121).

## 6 Limitations to the study

This scoping review is subject to several important limitations. First, the clinical evidence base mapping severe micronutrient deficiencies to pulmonary hypertension currently relies heavily on case reports and small, retrospective case series. By their inherent epidemiological nature, these study designs represent a low-to-moderate level of evidence. There is a stark contrast in evidence quality among the discussed pathways. While the prognostic weight of iron deficiency is well-established, particularly in adult cohorts and meta-analyses, similar epidemiological prevalence data and prognostic correlations for the other discussed vitamins remain strikingly absent. Due to the scoping nature of this review—which aims to chart emerging concepts and raise clinical awareness—we did not perform a formal risk of bias assessment (such as the JBI score), meaning the potential for reporting or publication bias cannot be excluded. Furthermore, while the basic science literature strongly supports the mechanistic pathways described, large-scale, prospective randomized controlled trials (RCTs) evaluating empirical metabolic optimization in these vulnerable pediatric cohorts remain entirely absent from the literature. Finally, overlapping biases related to co-existing morbidities, varying definitions of nutritional status, and unmeasured confounders in the included case reports preclude drawing definitive causal inferences regarding long-term prognostic outcomes. Furthermore, a notable limitation of universal oral pre-conditioning is the unpredictable enteral bioavailability in patients with decompensated right heart failure, where parenteral routes (IV/IM) remain mandatory to bypass mucosal edema and inflammatory blockades.

## 7 Conclusions

The clinical translation of the “Cellular Deprivation Model” within low- and middle-income countries (LMICs) necessitates a fundamental departure from Western-centric, diagnostic-heavy paradigms. In resource-constrained environments, the biochemical quantification of critical micronutrients is severely hindered by insurmountable logistical barriers. For instance, accurate plasma ascorbic acid determination requires immediate centrifugation, protection from light, and a strict −18 °C cold chain, while evaluating soluble transferrin receptor (sTfR) levels or thiamine status via high-performance liquid chromatography (HPLC) remains entirely unavailable in the vast majority of peripheral clinics.

Consequently, mandatory biochemical confirmation prior to initiating nutritional optimization introduces an unjustified and dangerous therapeutic delay. Because the cofactors analyzed in this review—namely vitamin C, thiamine, and folates—are water-soluble and possess exceptionally wide safety margins, and because physiological iron replacement is highly tolerated, a strategy of universal empirical metabolic pre-conditioning may represent the most pragmatic path forward. Rather than managing these deficits as ex-post targeted interventions, pediatric patients presenting with CHD or unexplained PH in epidemiologically vulnerable regions should receive a standardized multi-nutrient regimen, configuring metabolic optimization as a universal baseline therapy.

### 7.1 Call to action: a roadmap for pragmatic clinical trials

The international pediatric cardiology and global health communities should address this translational gap by designing and executing large-scale, prospective, randomized controlled trials (RCTs). The primary endpoints of these investigations should extend beyond standard somatic weight gain to incorporate hard hemodynamic and surgical outcomes.

Specifically, future trials should evaluate the efficacy of low-cost empirical supplementation protocols in:

- Improving right ventricular functional reserve (assessed via echocardiographic indices of systolic function such as TAPSE) and overall clinical tolerance (e.g., reducing feeding dyspnea in infants or improving functional capacity in older children) during the preoperative waiting period.
- Mitigating the incidence and severity of perioperative pulmonary hypertensive crises, as well as reducing postoperative mechanical ventilation times, by stabilizing the vascular endothelium and preventing eNOS uncoupling under cardiopulmonary bypass stress.
- Unmasking the reversible, “pseudo-fixed” vasoconstrictive component of pulmonary vascular resistance, thereby maximizing the reliability of preoperative vasoreactivity testing to more accurately stratify borderline patients for definitive vs. palliative surgical repair.
- Halting the progression of refractory cardiac cachexia, utilizing improved somatic growth trajectories as an indirect clinical marker of reduced resting energy expenditure and dampened hypoxia-driven (HIF-mediated) systemic inflammation.

Validating a standardized, low-cost metabolic protocol could fundamentally rewrite global health guidelines for managing structural and vascular heart diseases in impoverished settings. We provided a pragmatic framework for universal empirical supplementation in pediatric patients with structural heart disease or suspected metabolic PH within resource-limited settings, intentionally bypassing the need for advanced laboratory monitoring (Table 4).

**Table 4:** Empirical metabolic optimization protocol for pediatric pulmonary vascular disease in LMICs.

Micronutrient	Proposed empirical dose/nutritional target	Clinical red flags
Iron	Prophylaxis/Maintenance: 1–2 mg/kg/day orally. Empirical Treatment: 3 mg/kg/day orally (avoiding massive single doses to prevent hepcidin-mediated systemic blocking). In advanced right heart failure with gut edema, consider parenteral iron formulations (e.g., IV) if oral response is absent.	Progressive decline in exertional tolerance, worsening feeding dyspnea, or echocardiographic signs of right ventricular systolic failure. To be avoided in Thalassemia. In malaria-endemic regions, empirical supplementation must be paired with rapid diagnostic testing for parasitemia or adequate antimalarial coverage
Vitamin C (ascorbic acid)	Standard Empirical Dose: 100–200 mg/day orally (up to 1,000 mg/day intravenously during acute presentation).	Diets strictly limited to refined carbohydrates or boiled starches; unexplained gingival bleeding, petechiae, or lower-limb bone pain (subclinical scurvy).
Thiamine (vitamin B1)	Acute Intensive Care Rescue: 50–100 mg IV or IM immediately (in cases of decompensated shock), followed by maintenance therapy. Standard Empirical/Maintenance Dose: 5–10 mg/day orally.	Unexplained, progressive metabolic acidosis; hyperkinetic, high-output acute heart failure; fulminant sepsis-like syndromes with negative blood cultures.
Folates (B9), vitamin B12	Folic Acid: 5 mg/day orally. Vitamin B12: 1 mg/day orally (first seven days), 1 mg/day three times a week (2nd week), then 1 mg/once a week (Maintenance therapy).	Macrocytosis on a peripheral blood smear (regardless of frank hemoglobin reduction) or otherwise unexplained neurodevelopmental regression.
	In severe cases IM therapy (1 mg/day for 3 days) can be considered	

In patients presenting with decompensated right heart failure (characterized by severe hepatomegaly and systemic venous congestion), intestinal mucosal edema and inflammation-induced hepcidin spikes severely impair enteral absorption. In these acute scenarios, parenteral administration (IV/IM) must be prioritized to bypass the congested gastrointestinal tract and achieve effective metabolic rescue. IM, intramuscular; IV, intravenous; LMIC, low- and middle-income countries.
